# Experimental Flight Patterns Evaluation for a UAV-Based Air Pollutant Sensor

**DOI:** 10.3390/mi11080768

**Published:** 2020-08-11

**Authors:** João Otávio Araujo, João Valente, Lammert Kooistra, Sandra Munniks, Ruud J. B. Peters

**Affiliations:** 1 Information Technology (INF), Wageningen University (WUR), Hollandseweg 1, 6706 KN Wageningen, The Netherlands; joaootavio.araujodasilva@wur.nl; 2 Laboratory of Geo-Information Science and Remote Sensing, Wageningen University (WUR), Droevendaalsesteeg 3, 6708 PB Wageningen, The Netherlands; lammert.kooistra@wur.nl; 3 Wageningen Food Safety Research (WFSR), Akkermaalsbos 2, 6708 WB Wageningen, The Netherlands; sandra.munniks@wur.nl (S.M.); ruudj.peters@wur.nl (R.J.B.P.)

**Keywords:** unmanned aerial vehicle, electrochemical sensors, gas sensing, remote sensing

## Abstract

The use of drones in combination with remote sensors have displayed increasing interest over the last years due to its potential to automate monitoring processes. In this study, a novel approach of a small flying e-nose is proposed by assembling a set of AlphaSense electrochemical-sensors to a DJI Matrix 100 unmanned aerial vehicle (UAV). The system was tested on an outdoor field with a source of NO_2_. Field tests were conducted in a 100 m^2^ area on two dates with different wind speed levels varying from low (0.0–2.9m/s) to high (2.1–5.3m/s), two flight patterns zigzag and spiral and at three altitudes (3, 6 and 9 m). The objective of this study is to evaluate the sensors responsiveness and performance when subject to distinct flying conditions. A Wilcoxon rank-sum test showed significant difference between flight patterns only under High Wind conditions, with Spiral flights being slightly superior than Zigzag. With the aim of contributing to other studies in the same field, the data used in this analysis will be shared with the scientific community.

## 1. Introduction

Air quality is of growing concern over the past couple of decades and has led the United Nations (UN) as well as the European Union (EU) to elaborate a series of legislations [1] and packages [2] that target emission ceilings for pollutants. This issue is especially significant in Europe, where the pollution from one country affects the conditions of the others. That being so, it is crucial to improve local measures for monitoring air quality, additionally to maintaining international cooperation [3].

In the Netherlands, the National Institute for Public Health and the Environment (RIVM) is responsible for continuously measuring atmospheric indicators. This data is used to model air quality with great temporal resolution and is made publicly available [4,5]. According to the RIVM, air pollution has reduced over the past decades, but still every year 3000 people are affected by illnesses related to air pollution [4].

Respiratory problems have recently received a boost of attention from the media due to the 2019–2020 COVID-19 pandemic [6]. Even though further research is needed to address the relationship between air pollution exposure and vulnerability to this specific disease [7], it is already known that air contaminants, in general, are associated with other health disorders. Elevated concentrations of nitrogen dioxide (NO_2_), for example can affect liver, lung, spleen, blood and lead to respiratory issues [3]. This compound can also increase acidity and eutrophication of soil and water [3] and contribute directly and indirectly to air pollution as it is associated with the production of ozone (O_3_) [8].

In order to improve public health a more effective way of monitoring air quality is needed. Although the existing insights are valuable for temporal inference, there is potential for a more accurate spatial resolution of the problem [9]. With that information, areas that display higher pollution can be better pinpointed and thus prioritized. Much of the present mapping of volatiles is 2-dimensional and performed via satellite [10]. There is, however, an interest in a better understanding of the problem by the inclusion of altitude as a third dimension [11].

As the development of Unmanned Aerial Vehicles (UAVs) expands its availability, it also increases its interest for scientific research [12]. These vehicles have the potential to be a powerful tool in the field of gas sensing [13,14], as mobile measuring techniques are shown as a solution for obtaining data with improved spatial resolution [15]. Different from aircrafts or satellites [16,17], UAVs are a more flexible and potentially cheaper alternative that enables easy maneuvering at lower altitudes.

In combination with UAVs, small-sized sensors are expected to have a promising future, given that they present reliability in variable climatic conditions and capability of transferring data [18].

Mobile measuring tools, or active sensors as described by Neumann et al. [19], are based on the concept of actively searching for the plume of the target gas. Differently from static sensors that stay in position for long periods of time and perform measurements whenever gas plumes are carried close by the wind, mobile sensors are limited to their flight time, which could be around 20 min for an UAV. For that reason, flight path design is of extreme importance for optimal mobile sensing.

Because of the novelty of this field of study, there is still no agreement on the optimal design choices for active gas sensing (e.g., location of the sensor [20,21,22,23] and flight pattern [11,24,25]). Spiral and zigzag pattern were shown to have promising results for covering effectively the gas plume [24,25]. As for the sensor location, even though recommendations from Roldán et al. [22] and Villa et al. [23] are for top and side placement, respectively, in the current study they were positioned in the bottom of the UAV as it is common practice.

Although some studies investigated remote gas sensing strategies with sophisticated mobile platforms [20,24], while others used simpler and more available tools to study isolated design choices [26], the integration of such strategies and tools in a real-life outdoor situation lacks record. There is still a lot of uncertainty regarding the effect of natural factors in the performance of mobile gas sampling. Finally, currently there are no public datasets combining UAV and electrochemical data. This would significantly strength the research in this domain.

The present study aimed at evaluating the combination of geometric flight pattern [27] and altitude in an outdoor situation with wind as an environmental factor of influence.

The role of wind in such measurements is recognized by previous researchers [20,24,25] as a determinant factor for planning the flight path. The authors however are not in consensus regarding the severity of the wind.

## 2. Materials and Methods

### 2.1. Unmanned Aerial Vehicle(UAV) Set-up

The UAV used during the experiments was a DJI Matrice 100 (Shenzhen, China), which can easily have new sensors attached to it, making it advantageous for research. The set-up was developed by ROBOR Electronics B.V., a company specialized in wireless sensors, in collaboration with Wageningen Food Safety Research (WFSR) that also owns the UAV.

**Figure 1:**
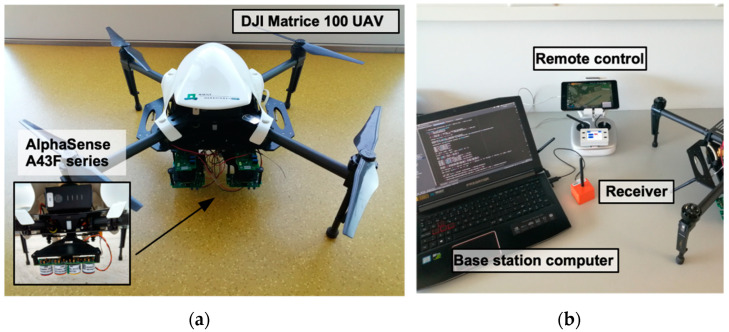
Unmanned Aerial Vehicle (UAV)-set up: (**a**) A top view of the DJI Matrice 100 UAV used during the experiments and a focus on the mounting point of the A43F series sensors, located underneath the drone. (**b**) The ground station, where the UAV was controlled and data was gathered and stored.

In total, eight sensors were attached to the UAV: four AlphaSense A43F (Essex, United Kingdom) series sensors for NO_2_, Cl_2_, H_2_S and SO_2_, additionally to four smaller sensors TGS2600, TGS2602, TGS2610, TGS2620 (Arlington Heights, IL, USA) for general air contaminants and volatile organic compounds (VOCs). Only the NO_2_ sensor was used during the present study. Figure 1 illustrates the UAV set-up. Data gathered by the sensor was obtained semi-real time through a wireless connection with a computer at the ground station.

The way that AlphaSense electro-chemical sensors work is based on four electrodes. Two of these are called Working Electrode (WE) and Auxiliary Electrode (AE). The WE performs a redox reaction with the target gas, resulting in a current which measures its concentration, while the AE works as a calibration, correcting the measurements from the WE. Henceforth, the NO_2_ measurements will also be referred as WEAE.

WEAE values can be converted to realistic NO_2_ concentrations (µg m^−3^) through Equation (1), based on Mijling et al. (2018) [28]:

(1) $$NO_{2}=c_{0}+c_{1}*S_{WE}+c_{2}*S_{AE}+c_{3}*T+c_{4}*RH$$

which could be further improved with the addition of a factor that accounts for cross-sensitivity with O_3_. In this research, conversion for NO_2_ concentration was not performed, because the analysis was based on a proportion of maximum measurement in each trial, which are not expected to be affected by changes in temperature and relative humidity in such a short flying time of each trial.

Flight autonomy of the UAV was estimated to 160 min, provided by eight batteries that contributed to 20 min each.

### 2.2. Experimental Design

In order to evaluate the effect of different design choices on the performance of NO_2_ mobile measurement, flight patterns (spiral and zigzag) were recombined with different flying heights and repeated in two days with different weather conditions as summarized by Table 1.

**Figure 2:**
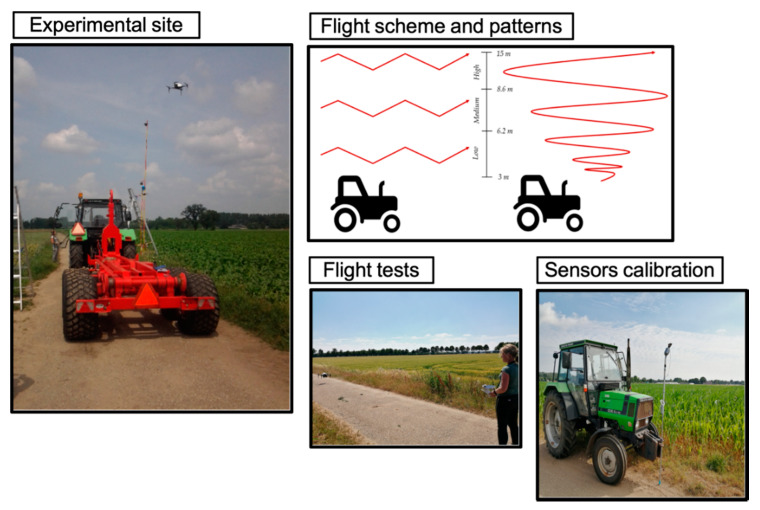
Experimental setup: Experimental site where the experiments were carried out; Flight scheme and patterns (zigzag and spiral); Flight tests for testing the sensors and communication with the base station; Sensors calibration for setting a threshold for the measurements.

The experiments were conducted in an open field that belongs to Wageningen University & Research, with no nearby obstructions for the wind flow. An artificial pollutant plume was generated with constant output by the combustion of fuel from the 3.3 L diesel engine of a tractor Deutz Fahr DX4.11. SE. Above this plume, the UAV was flown several times for each flight pattern with varying altitudes as illustrated by Figure 2.

### 2.3. Flight Patterns

The zigzag pattern consisted of flying 15 m starting next to the source in the direction of the wind while performing 60° turns to ensure a large coverage of the area. This procedure was based on the research of Neumann et al. [25], Kim et al. [24] and Berg et al. [20]. Spirals were performed at a starting altitude, rising 7 m while expanding the spiral and ending up with a radius of 15 m. This action was done next to the artificial emission source, going upwards and slowly increasing the spiral radius. The work of Peng et al. [29] was used as reference for this pattern.

### 2.4. Data Pre-Processing

**Figure 3:**
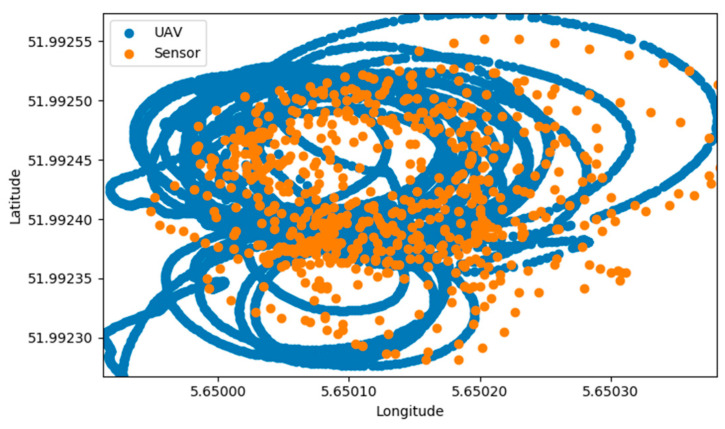
Latitude and Longitude coordinates plot of the UAV and Sensor’s GPS, indicating much lower resolution of the sensor.

Because the precision of the UAV’s GPS was much greater than that of the gas sensor (Figure 3), a calibration of the data had to be done. By using the timestamp, gas measurement values were synchronized with their respective corrected coordinates of latitude and longitude. Due to the data acquisition rate of the UAV being at every decimal of a second instead of every second as of the gas sensor, WEAE values were copied to every position that corresponded to the same integer of seconds.

The altitude of flight was aimed to be close to the heights where the static sensors were positioned, but during data pre-processing it was shown that the drone flew over a way more varied range of altitude. Consequently, the altitude ranges considered for data analysis were estimated by dividing the whole data set into terciles for Low, Medium and High altitude levels (Alt Level) as shown in Table 2.

Another categorization used was with regards to the measured gas, limiting the range of this output from 0 to 1. As a way to standardize the results, gas sensing outputs were normalized and categorized separately for each flight trial. In this way, each trial had its own Low, Medium, and High WEAE levels (WEAE Level: Table 2).

Each trial consisted of a series of flights using the same pattern, performed at different altitudes and during the same day, thus same wind speed level. The separation in trials was done due to the capacity of the batteries.

Individually normalization and classification of WEAE outputs were meant to reduce the influence of uncontrolled factors of the experiment, such as changes in temperature, relative humidity, or wind direction between each different trial. It is expected that these environmental factors will not present major shifts within the timespan of each trial (12 min and 30 s maximum) but may be significant when comparing the first and last trial of the day (3 h apart). Furthermore, normalization reduced the effect of outliers when the UAV passed too close to the emission source. Finally, parts of the trials needed to be cropped out after identifying moments when the drone was being maneuvered or simply when it was static due to recharge.

## 3. Results

**Figure 4:**
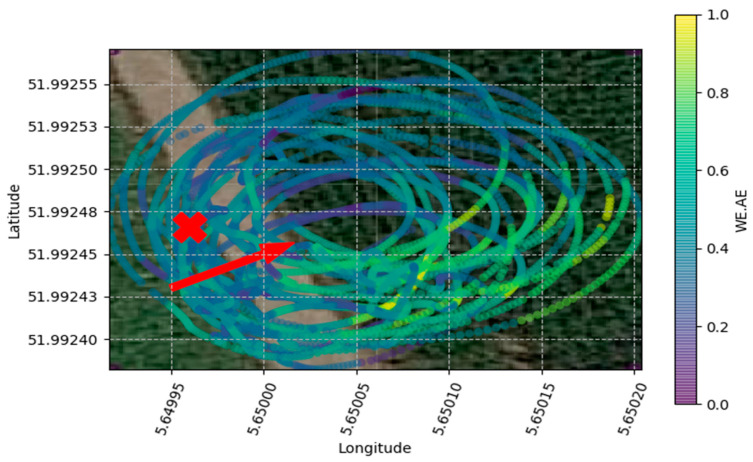
Variation of NO_2_ during the spiral flight trial executed during the high wind day. Background satellite image was acquired from Google database. The red X indicates the position of the NO_2_ source and the red arrow indicates wind direction.

Each flight trial was observed individually along with a color scale to help visualize possible trends in the distribution of the measured gas (Figure 4).

### 3.1. Flight Pattern

**Figure 5:**
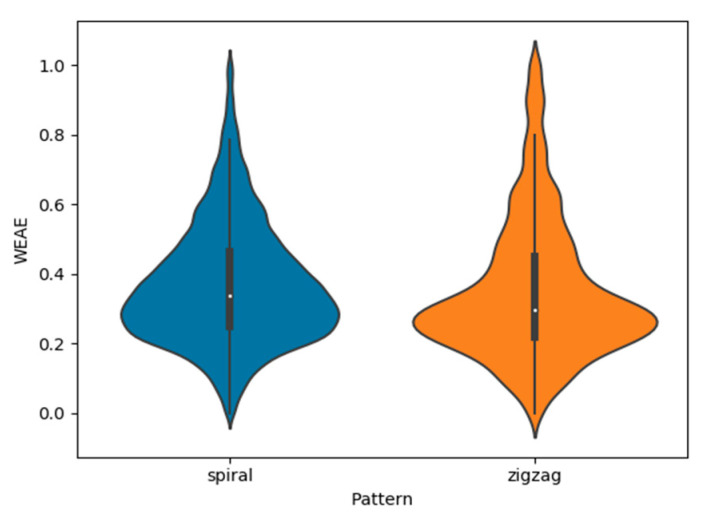
Density distribution of normalized NO_2_ outputs with flight pattern as the only discriminating factor, illustrated by a violin plot.

Because the outputs of each trial were normalized, the analysis method used for this research consisted of observing the influence that each factor had on the distribution of the gas measured, not in absolute values. To support a more informative visualization of the distribution, violin plots were used instead of regular boxplots, as the first is capable of expressing the density of each value of interest with more detail than the latter [23]. When comparing both flight patterns without discrimination of wind condition or altitude, the shape of the distribution seemed rather similar, with Low to Medium levels of WEAE composing the largest density of measured points, decreasing steadily for higher levels of the gas (Figure 5). No other local maxima or minima were identified apart from the already indicated Low to Medium band.

By separating the violin plots of gas measurements for each wind level (one for each day of experiments), it becomes noticeable that each of them present a specific trend for the shape of the distribution, while within each wind level, the patterns hardly differentiate between themselves. High Wind shows more evenly distributed results when compared to Low Wind, which presents a visible concentration of WEAE values between the range 0.2–0.4 and low density of values greater than 0.5.

Average WEAE for Low Wind for spiral and zigzag were 0.28 and 0.26, respectively. While for High Wind, these values increased to 0.42 for the spiral pattern and 0.45 in the case of zigzag.

### 3.2. Wind Speed

**Figure 6:**
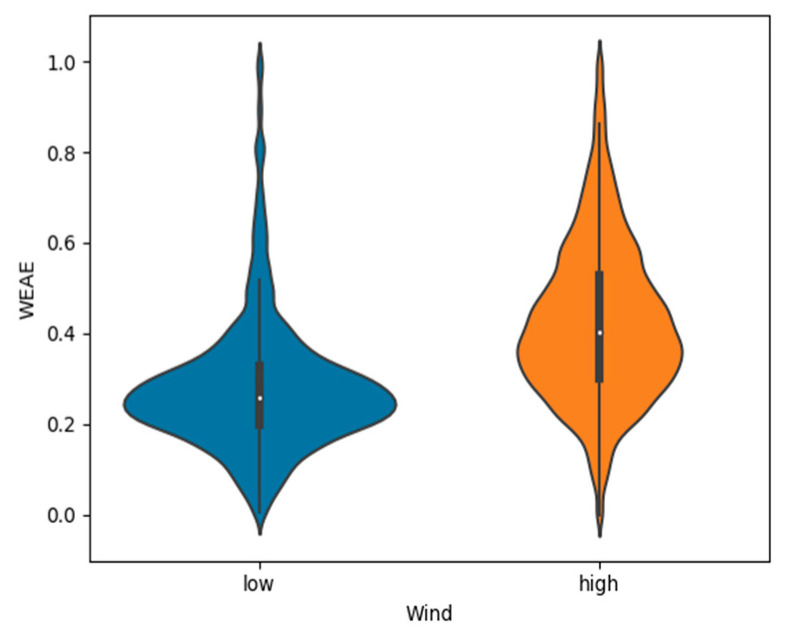
Comparison of WEAE distribution for all flight patterns and altitudes flown, with wind speed as discriminant factor. Low Wind conditions present high density of medium to low values of gas concentration measurements, while High Wind yields more evenly distributed ones and a higher average.

Due to the distribution shape of flight patterns be similar within each wind level, but different between them, a next step would be to compare results accounting for this environmental factor. As seen from Figure 6, the distribution shape is much more uniform for a High Wind condition, meaning that sampling with such conditions lowers the occurrence of outliers and ensures a larger sample size for all the range of measurements.

**Figure 7:**
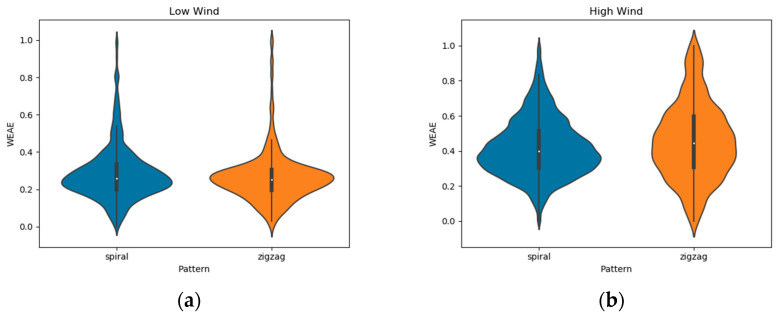
Influence of each flight pattern separated between: (**a**) Low Wind, gathered on June 15, and (**b**) High Wind, gathered on June 20.

The effect of wind speed on the shape of distribution maintains for each individual flight pattern analysed. Figure 7 indicates that a more homogeneous distribution of measurements under higher wind speed is constant regardless of the pattern of flight used.

### 3.3. Altitude

**Figure 8:**
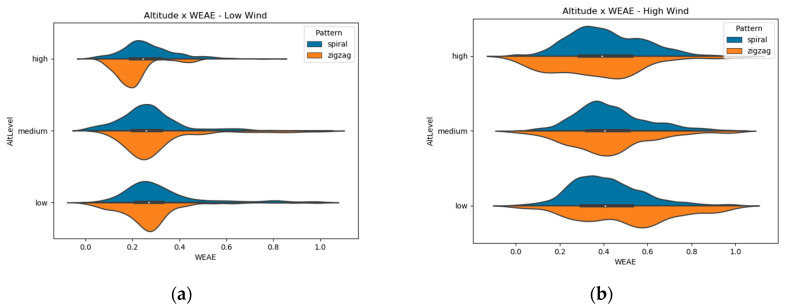
Violin plots of gas measurements at each layer of altitude for (**a**) Low Wind conditions and (**b**) High Wind conditions.

The violin plots of altitude show that the same shape observed on the different wind levels are maintained at every level of altitude (Figure 8).

**Figure 9:**
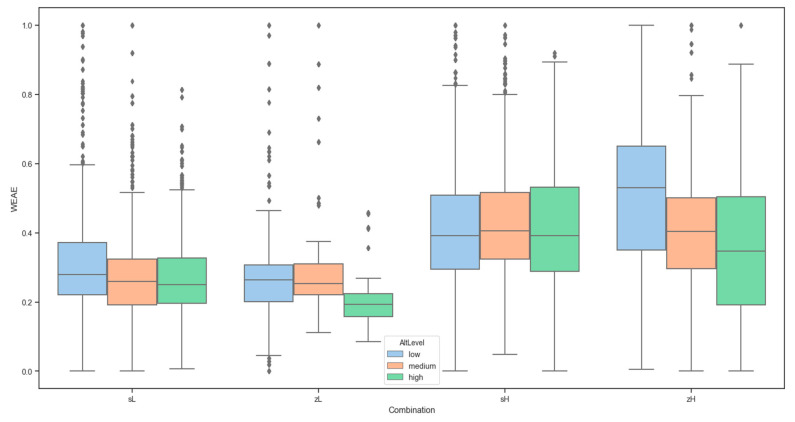
Gas measurement relative values according to each combination of flight path: spiral + Low wind (sL), spiral and High wind (sH), zigzag and Low wind (zL) and zigzag and High wind (zH).

A boxplot (Figure 9) with each combination of pattern (spiral or zigzag) and wind conditions (Low or High) separated for altitude levels (Low, Medium and High) showed similar averages between treatments.

## 4. Discussion

During data analysis, the initial hypothesis was that spiral flights would yield more consistent results, with a uniform distribution of the outputs and a clear range of values that would present higher density. This was due to the longer flight time that this pattern required to perform its trajectory, which yielded almost 5 times more data points that zigzag flights.

However, results showed that both flight patterns barely differentiated from each other with respect to the density of measurements (Figure 6). Spiral flights were able to capture slightly more *Medium*-*High* gas concentration relative to zigzag ones, but still not enough to produce a uniform distribution of the range of gas concentrations the sensor is able to perceive.

Kolmogorov-Smirnov tests for goodness of fit were performed to check the uniformity of the data sets. All tests resulted in non-uniformity for the four possible combinations of Wind Level and Pattern. However, the highest p-value was attained for High Wind conditions and Zigzag pattern.

While zigzag pattern outliers could have been due to the shorter flight time, as initially hypothesized, the occurrence of these high values in spiral flights could have been a consequence of starting the flight pattern close to the artificial gas source.

Although flight pattern influence in gas sensing performance was not of great significance, wind speed during the measuring day was the factor with the most impact in the present study. The wind effect is mainly present in the shape of the gas plume, affecting its dispersion and shape. Many gas dispersion models take wind into consideration as it is responsible for molecular diffusion and transportation of gases [30]. A higher wind speed might have caused a broader dispersion of NO_2_, causing the results to be more homogeneous. Other environmental factors that differed between the experimental dates, such as temperature and relative humidity did not present as much of a variation as wind did, and so this factor was considered to be the most influential one. Average temperature and humidity resulted in a variation of 20 and 13%, respectively, while wind almost doubled its maximum value.

Furthermore, due to the lack of normality of the data, a Wilcoxon rank-sum test was performed between flight patterns of the same wind speed level to check if they differed significantly. The test was performed considering low levels of NO_2_ measurements only, in order to avoid outliers. The outcome of the test showed that in Low Wind, patterns did not differentiate (*P* = 0.538), while at High Wind, the average of Spiral flights was significantly higher than that of Zigzag ones (*P* = 0.0002). The mentioned distributions can be observed in Figure 7.

Altitude, as flight pattern, showed no significant changes in the distribution shape of NO_2_ relative concentration measurements. Previous studies [22,23] pointed to the fact that the wind produced by the rotors might blow the wind from above downwards, causing the concentration of the gas to be mixed between different altitudes. This assumption needs to be studied further.

A new point of attention that emerged from the analysis of altitude data was with regards to the different directions of gas dispersion. Gas is also transported not only upwards, but also horizontally, meaning that the distance from the artificial source could have influenced the results. In order to observe the effects of horizontal dispersion, a convex hull function was used to determine the center of each flight trial according to the perimeter of the polygon formed by the coordinates of all points in a trial. The center of each polygon was calculated and the distance of each measurement to this point was estimated (Center Distance) according to latitude and longitude coordinates.

**Figure 10:**
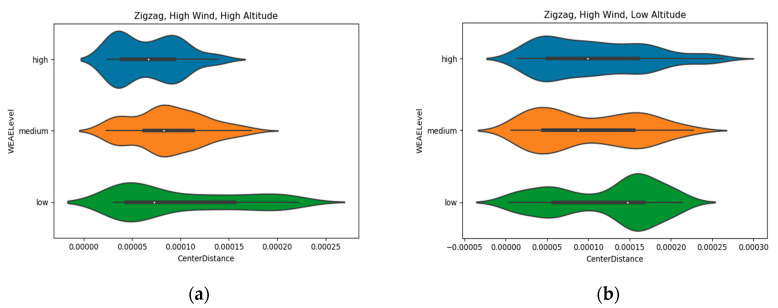
Occurrences of different gas measurements concentration levels at (**a**) high and (**b**) low altitude compared to distance of the location of the measurement and the center of the area explored, where the gas source was located.

Another violin plot was used to analyze the influence of the previously mentioned dispersion. The results (Figure 10) point to a vanishing occurrence of high concentration measurements as the coverage area gets wider in high altitudes, while at low altitudes high concentration data points are present over an even wider range from the center than low concentration ones.

A multiple linear regression was attempted for explaining gas measurements based on Wind Level, Altitude, Pattern and Center Distance, as done in the work of Villalobos and Fereres [26]. However, the low value of the adjusted R-squared (0.172) suggests that the model’s parameters present low explanatory value. For future works, temperature and relative humidity should also be measured during the flight trials, as these parameters are found to increase the accuracy of multilinear regression applied for NO_2_ sensors calibration [28]. The initial calibration attempted for the present study was not successful due to the need of extra material and the short time available to perform this task during the conduction of the experiments.

## 5. Conclusions

This study aimed at evaluating two strategical factors (flight pattern and altitude) and one environmental factor (wind) on the performance of NO_2_ gas measurement using a mobile platform (Video S1). Influence of strategy factors, such as flight pattern and altitude, as well as environmental factors, such as wind speed are not enough to determine all the variations in the performance of a gas sensing technology using UAVs in an outdoor environment. Some factors not studied in this research, such as temperature and relative humidity surely present their influence and depend on a better calibration method to be taken into account.

From the factors analyzed, wind speed showed to have caused higher variance between treatments. To ensure if this effect was large enough to have overwhelmed the strategy factors, a study done in a controlled environment with more gradually changing and controlled wind is advised.

The results acquired during this study indicate a zigzag pattern at high wind speeds as the most promising combination of factors to result in a more homogeneous gathering of data. High wind choice is based in the more evenly distributed output from this condition. For flight pattern, zigzag is preferred over spiral because both patterns demonstrated no significant differences in distribution shape, but zigzags require less flight time and consequently less battery, grating the possibility to perform a higher number of flights before recharging.

Furthermore, the data sets used in this research will be made available to the scientific community in order to promote research in the field of remote sensing with combined systems of UAV and gas measuring tools. The datasets can be accessed in https://git.wur.nl/said-lab/drone-olfaction.git.

## Figures and Tables

**Table 1:** Environmental conditions summary of the experiment days.

Day	Start	End	Temperature	Humidity	Wind Speed	Wind Direction
June 15	09:54	14:01	24 °C	70%	0.0–2.9 m s^−1^	NE
June 20	09:35	13:06	20 °C	79%	2.1–5.3 m s^−1^	NE

**Table 2:** Summary of the experimental ranges obtained after categorization of gas measurements (WEAE Level) and altitude (Altitude Level). Separation of the data was done with the function qcut from the Pandas library in Python.

Trial	Wind Level (m·s^−1^)	WEAE Level (0–1)			Altitude Level (m)		
		Low	Medium	High	Low	Medium	High
Spiral 1	Low (0.0–2.9)	0.0–0.26	0.27–0.38	0.39–1.0	3.0–6.2	6.3–8.6	8.7–15.0
Spiral 2		0.0–0.23	0.23–0.29	0.29–1.0			
Spiral 3		0.0–0.19	0.19–0.25	0.26–1.0			
Zigzag 1		0.0–0.17	0.17–0.25	0.26–1.0			
Zigzag 2		0.0–0.26	0.26–0.30	0.30–1.0			
Spiral 1	High (2.1–5.3)	0.0–0.35	0.35–0.48	0.48–1.0			
Spiral 2		0.0–0.28	0.28–0.38	0.38–1.0			
Spiral 3		0.0–0.41	0.41–0.54	0.54–1.0			
Zigzag 1		0.0–0.30	0.30–0.50	0.51–1.0			
Zigzag 2		0.0–0.25	0.25–0.35	0.39–1.0			
Zigzag 3		0.0–0.44	0.44–0.62	0.63–1.0			
Average		0.0–0.28	0.29–0.39	0.40–1.0			

## Supplementary Materials

The following are available online at https://www.mdpi.com/2072-666X/11/8/768/s1, Video S1: Flight Patterns Evaluation for a UAV-Based chemical foot printing.
